# Left Cardiac Sympathetic Denervation as an Acute Treatment of Torsades in a Paediatric Case of Long QT

**DOI:** 10.1016/j.cjcpc.2022.10.008

**Published:** 2022-11-03

**Authors:** Jennifer Shortland, Diego Marquez, Osman Al-Radi, Shubhayan Sanatani

**Affiliations:** a Division of Cardiology, Department of Pediatrics, University of British Columbia, Vancouver, British Columbia, Canada; b Division of Pediatric Cardiothoracic Surgery, Department of Surgery, University of British Columbia, Vancouver, British Columbia, Canada

**Congenital long QT syndrome (LQTS) is characterized by abnormal cardiac repolarization with a heart rate corrected QT prolongation and life-threatening arrhythmias leading to syncope and sudden death (Tian et al. *Circ Arrhythm Electrophysiol* 2019;12:e007118). Thoracoscopic left cardiac sympathetic denervation is a surgical antiadrenergic intervention used for primary and secondary prevention in inherited arrhythmia syndromes. It is a safe and effective treatment option for LQTS patients with medically refractory arrhythmias, medication intolerance or contraindication, or a history of appropriate implantable cardioverter-defibrillator shocks (Collura et al. *Heart Rhythm* 2009;6:752-9). We report on its use in paediatrics as an acute therapy for refractory ventricular arrhythmias in a case of gene-elusive LQTS**.

## Case Report

A 16-year-old, previously healthy girl presented to the emergency department (ED) with a reduced level of consciousness. She had gone to sleep as usual, but was found to be difficult to rouse by her mother in the morning. She was drowsy with incoherent speech and was unable to stand without assistance. It was noted that she had been incontinent of urine. On arrival at the ED, the patient had a Glasgow Coma Scale of 8. Her initial 12-lead electrocardiogram (ECG) demonstrated sinus rhythm with a corrected QT interval of 530 milliseconds (ms). Extended serum electrolytes and blood glucose were normal. A computed tomography head was normal. She received 1 mg of sublingual lorazepam before the computed tomography and within 2 hours after arrival at the ED; her Glasgow Coma Scale had normalized. One hour after return of normal cognition, the patient developed a sustained, regular wide complex, and monomorphic tachycardia at a rate of 180 beats per minute (bpm) with no haemodynamic compromise, and she remained cognizant. At the adult hospital where she initially presented, she received ketamine sedation and underwent 3 direct current cardioversions with return of sinus rhythm. Because of the wide complex tachycardia in the context of a prolonged QTc, an intravenous magnesium sulphate bolus was given. On direct questioning, the patient reported episodic palpitations that occurred every few months for the past 2 years. These were associated with symptoms of fatigue and self-resolved after a few minutes. There was no family history of congenital heart disease, sudden death, or arrhythmias. She denied any regular medications, and a full urine toxicology screen was negative including cannabinoids. There were no recent infectious contacts, and COVID testing was negative.

**Novel Teaching Points**

• This case report highlights the potential use of LCSD as an acute therapy in paediatric patients with LQTS for the treatment of β-blocker–resistant ventricular arrhythmias and should be considered before the placement of an ICD particularly in patients who are at high risk for an electrical storm.

**Figure 1:**
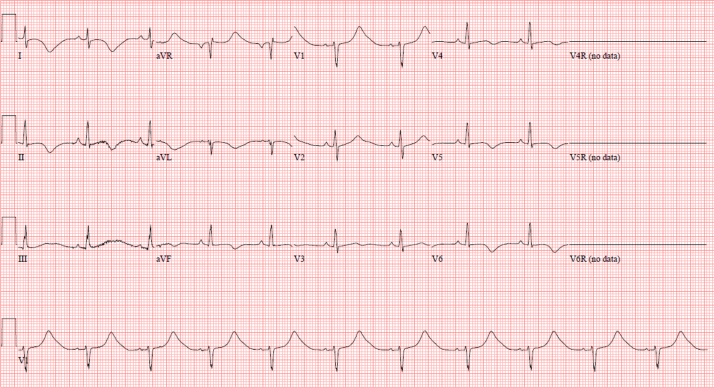
Twelve-lead electrocardiogram demonstrating sinus rhythm at a rate of 65 bpm. There is widespread abnormal repolarization with T-wave inversion in the lateral and inferior leads. The T waves are broad based, and the QTc is significantly prolonged at 584 ms.

**Figure 2:**
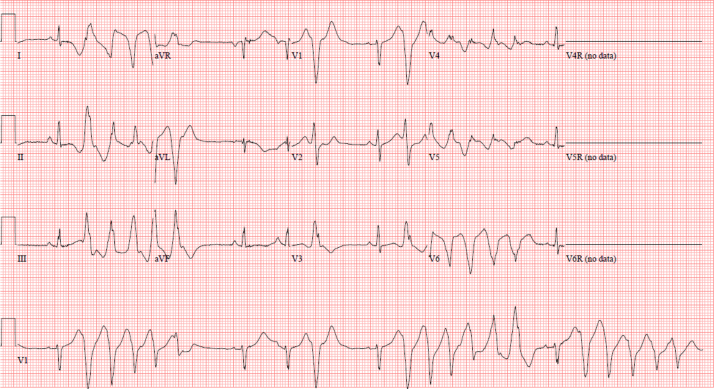
Twelve-lead electrocardiogram demonstrating 2 runs of torsades de pointes, which is characterized by a wide complex polymorphic ventricular tachycardia.

Subsequent ECGs showed sinus bradycardia, a QTc of 584 ms, and abnormal repolarization with inverted T waves in the left lateral precordial leads (Fig. 1). Left ventricular systolic function was mildly decreased on the initial echocardiogram but subsequently quickly normalized. The following morning, the patient developed a high ectopy burden of ventricular bigeminy and trigeminy and was commenced on intravenous esmolol at 50-75 mcg/kg/min and oral propranolol 3.2 mg/kg/d divided into 3 doses. This resulted in significant sinus bradycardia to 35 bpm and increasingly prolonged runs of complex ventricular ectopy including runs of torsades de pointes (TdP) (Fig. 2). An isoproterenol infusion was initiated and increased to a maximum of 0.15 mcg/kg/min, resulting in an increased heart rate of 50-85 bpm and a reduction in the frequency of TdP.

Oral spironolactone of 25 mg daily for 48 hours was started on day 2 of admission and trialed to optimize serum potassium levels, and the patient received several intravenous boluses of magnesium sulphate. Long QT type 2 was suspected because of the clinical presentation, which was presumed to be secondary to a nocturnal arrhythmia causing cerebral hypoperfusion leading to seizure and subsequent postictal confusion. Mexiletine was therefore added on day 3 of admission at 100 mg twice daily for 24 hours; a relatively small dose was selected to ensure gastrointestinal tolerance. The patient remained asymptomatic despite frequent salvos of TdP.

Despite maximally tolerated doses of β-blockers and the addition of mexiletine and spironolactone, the patient continued to experience short runs of TdP on the attempted reduction of the isoproterenol. The potential of electrical storms in this patient was high if an ICD was implanted without additional antiarrhythmic intervention. Therefore, thoracoscopic left cardiac sympathetic denervation (LCSD) was performed acutely in conjunction with ICD placement.

The patient underwent thoracoscopic LCSD 3 days after her admission. The procedure was performed under general anaesthetic. The sympathetic chain was dissected from T5 to the stellate ganglion, and the lateral branches were divided. After completion of the dissection, the sympathetic chain was divided at the level of T5. The entire sympathetic chain was resected en bloc from T5 to the midportion of the stellate ganglion.

After the sympathetic denervation, the patient underwent insertion of an endocardial dual chamber ICD with a Medtronic Evera MRI S DR SureScan generator. A defibrillation threshold test, as per institutional practice, was successful. Settings were programed at AAI-DDD, rate between 60 and 130 ppm.

A repeat ECG 2 days after the procedure showed an underlying rhythm of sinus bradycardia at a rate of 59 bpm and upright T waves throughout all precordial leads. There was also a reduction in the corrected QT interval to 476 ms. Mexilitine and spironolactone were discontinued postoperatively, and she was discharged home 3 days after the procedure on oral propranolol. The patient experienced transient numbness at the upper chest and flank 2 weeks after the procedure but no other adverse effects. A comprehensive 217 gene cardiology genetic panel was negative for a known inherited cause of long QT. Thirteen months after her procedure, the patient’s QTc has been measured at a maximum of 480 ms, and on recent exercise testing, her repolarization remained borderline throughout with a QTc of 457 ms at 4-minute recovery. There have been no ventricular arrhythmias recorded and no ICD therapies delivered.

## Discussion

Congenital long QT syndrome (LQTS) is a life-threatening cardiac arrhythmia syndrome characterized by ventricular tachyarrhythmia, syncope, and sudden death.^1^ This syndrome represents a leading cause of death in the young. There are at least 13 known LQTS genes; however, KCNQ1 (LQT1), KCNH2 (LQT2), and SCN5A (LQT3) are the most common and account for around 90% of all genotype positive cases.^2^ Electrocardiographic signs include a prolonged corrected QT interval and morphologic abnormalities of ventricular repolarization including T-wave notching or alternans. Arrhythmogenic episodes include TdP, which, if transient, can result in syncope or can degenerate into ventricular fibrillation causing cardiac arrest. Among untreated patients, the mortality rate is high—21% within 1 year of the first episode of syncope.^2^

Our patient’s presentation was unusual for congenital LQTS. Her altered level of consciousness in the ED was not initially felt to be cardiac in etiology as her rhythm throughout was sinus. This resolved over the course of several hours and after the administration of lorazepam. We suspect that the patient had a nocturnal episode of ventricular arrhythmia resulting in cerebral hypoperfusion and resulting in her presentation with altered consciousness. The initial episode of sustained monomorphic ventricular tachycardia was also unusual, and our patient continued to have nonsustained runs of polymorphic ventricular tachycardia before the LCSD. All ECGs before intervention demonstrated a significantly prolonged corrected QT interval and intractable ventricular arrhythmias, with no evidence of an acquired cause. Antiarrhythmic control was not achieved despite β-blockers, sodium channel blockers, or nonselective β-adrenoceptor agonists, but maximal dosing was not always achieved because of a concern for side effects or continuing rhythm instability necessitating more urgent invasive management. Our centre does not have experience with percutaneous temporary LCSD, and so the decision was made to perform an LCSD as the primary antiarrhythmic therapy, as the only option to potentially alter the intrinsic QT interval. The LCSD procedure was well tolerated with no long-term side effects and effective, leading to complete remission of her ventricular arrhythmias.

The internationally accepted management of symptomatic patients with LQTS recommends initial management of lifestyle modifications and the commencement of β-blocker therapy. Long-acting β-blockers such as nadolol or propranolol are shown to be more effective at reducing breakthrough cardiac events.^3^ Device therapy in patients with LQTS still remains a class I indication for patients with LQTS who have an out-of-hospital arrest but is recognized to have significant disadvantages in the paediatric population such as inappropriate shocks and device-related complications.^4^

The effectiveness of LCSD in LQTS was first proposed after pioneering studies by Yanowitz and Schwartz demonstrated the arrhythmogenic properties of the left stellate ganglion. This procedure was first used as a therapeutic treatment for LQTS by Arthur Moss over 50 years ago. It is now internationally recognized as an effective treatment for drug refractory ventricular arrhythmias in the channelopathies LQTS and catecholaminergic polymorphic ventricular tachycardia and recommended by the HRS/EHRA/APHRS consensus for use in high-risk LQTS patients in whom ICD therapy is contraindicated or refused or in patients where β-blocker therapy is not successful at preventing syncope, not tolerated, refused, or contraindicated.^3^ The antiarrhythmogenic mechanism of LCSD is thought to be due to a combination of the prevention of norepinephrine release in the ventricles from the left-sided cardiac nerves, increased threshold for ventricular fibrillation, increased ventricular refractoriness, and its prevention of alpha-adrenergic–mediated increases in repolarization heterogeneity caused by early and delayed repolarizations. Importantly, it does not decrease heart rate or impair left ventricular function, and the procedure is not followed by reinnervation or postdenervation supersensitivity.^5^

This procedure is not without complications, including left-sided dryness, unilateral facial flushing, contralateral hyperhidrosis, and differential hand temperatures among the most commonly reported.

The successful use of percutaneous left stellate ganglion block as an acute therapy for refractory arrhythmias has also been reported. A systematic review of 38 adult patients who presented with electrical storms demonstrated a significant reduction in the burden of ventricular arrhythmias and the number of external or implantable cardioverter-defibrillator shocks administered after unilateral percutaneous local anaesthetic administration to the stellate ganglion.^6^ This effect was temporary and the duration of the suppressive effect varied depending on the anaesthetic agent used. The use of LCSD as an acute treatment in paediatric electrical storm is not commonly performed.

This procedure was successful in our patient leading to remission of her ventricular arrhythmias. In some LQTS patients, temporary atrial pacing (transesophageal or transvenous) could be considered acutely to increase the heart rate and potentially shorten the QT interval. Percutaneous stellate ganglion block could also be considered as a temporizing method or to guide management if performed in a centre experienced with this technique.

## References

[1] Brugada J., Blom N., Sarquella-Brugada G. (2013). Pharmacological and non-pharmacological therapy for arrhythmias in the pediatric population: EHRA and AEPC-Arrhythmia Working Group joint consensus statement. Eurospace.

[2] Schwartz P., Crotti L., Insolia R. (2021). Long-QT syndrome: from genetics to management. Circ Arrhthym Electrophysiol.

[3] Prior S., Wilde A., Horie M. (2013). HRS/EHRA/APHRS expert consensus statement on the diagnosis and management of patients with inherited primary arrhythmia syndromes. Heart Rhythm.

[4] Olde Nordkamp L.R.A., Postema P.G., Knops R.E. (2016). Implantable cardioverter-defibrillator harm in young patients with inherited arrhythmia syndromes: a systematic review and meta-analysis of inappropriate shocks and complications. Heart Rhythm.

[5] Dusi V., Pugliese L., De Ferrari G. (2022). Left cardiac sympathetic denervation for long QT syndrome: 50 years’ experience provides guidance for management. JACC Clin Electrophysiol.

[6] Meng L., Tseng C., Shivkumar K., Ajijola O. (2017). Efficacy of stellate ganglion blockade in managing electrical storm. A systematic review. JACC Clin Electrophysiol.

